# Identifying the relative importance of predictors of survival in out of hospital cardiac arrest: a machine learning study

**DOI:** 10.1186/s13049-020-00742-9

**Published:** 2020-06-25

**Authors:** Nooraldeen Al-Dury, Annica Ravn-Fischer, Jacob Hollenberg, Johan Israelsson, Per Nordberg, Anneli Strömsöe, Christer Axelsson, Johan Herlitz, Araz Rawshani

**Affiliations:** 1 University of Gothenburg, Institute of Medicine, Sahlgrenska Academy, Gröna Stråket 4, 43146 Gothenburg, Sweden; 2 Department of Radiology, Østfold Hospital Kalnes, Grålum, Norway; 3 Department of Cardiology, Sahlgrenska University Hospital, Gothenburg, Sweden; 4 Department of Medicine, Center for Resuscitation Science, Karolinska Institutet, Solna, Stockholm, Sweden; 5 Division of Cardiology, Department of Internal Medicine, Kalmar County Hospital, Kalmar, Sweden; 6 Kalmar Maritime Academy, Linnaeus University, Kalmar, Sweden; 7 Department of Cardiology, Södersjukhuset, Stockholm, Sweden; 8 Center for Resuscitation Science, Karolinska Institutet, Stockholm, Sweden; 9 School of Health, Care and Social Welfare, Västerås, Sweden; 10 Centre for Prehospital Research, Faculty of Caring Science, Work Life and Social Welfare, Borås, Borås, Sweden

## Abstract

**Introduction:**

Studies examining the factors linked to survival after out of hospital cardiac arrest (OHCA) have either aimed to describe the characteristics and outcomes of OHCA in different parts of the world, or focused on certain factors and whether they were associated with survival. Unfortunately, this approach does not measure how strong each factor is in predicting survival after OHCA.

**Aim:**

To investigate the relative importance of 16 well-recognized factors in OHCA at the time point of ambulance arrival, and before any interventions or medications were given, by using a machine learning approach that implies building models directly from the data, and arranging those factors in order of importance in predicting survival.

**Methods:**

Using a data-driven approach with a machine learning algorithm, we studied the relative importance of 16 factors assessed during the pre-hospital phase of OHCA. We examined 45,000 cases of OHCA between 2008 and 2016.

**Results:**

Overall, the top five factors to predict survival in order of importance were: initial rhythm, age, early Cardiopulmonary Resuscitation (CPR, time to CPR and CPR before arrival of EMS), time from EMS dispatch until EMS arrival, and place of cardiac arrest. The largest difference in importance was noted between initial rhythm and the remaining predictors. A number of factors, including time of arrest and sex were of little importance.

**Conclusion:**

Using machine learning, we confirm that the most important predictor of survival in OHCA is initial rhythm, followed by age, time to start of CPR, EMS response time and place of OHCA. Several factors traditionally viewed as important, e.g. sex, were of little importance.

## Introduction

Out of hospital cardiac arrest (OHCA) carries a dismal prognosis. The overall survival after OHCA in Sweden during 2008 to 2016 was approximately 10% [1], and similar numbers are noted in the US [2]. Some of the most important factors linked to survival after OHCA include initial rhythm, bystander Cardiopulmonary Resuscitation (CPR) and defibrillation, ambulance response times, age, sex, location, and cause of OHCA [3–13]. Most studies examining these factors have employed traditional regression models to describe the association between various characteristics and survival. Although such an approach does offer important insights, it does not formally assess the relative importance of each factor to predict survival after OHCA. Moreover, the traditional approach to regression modelling implies building models from theory and subject matter knowledge, which is prone to bias via subjective preferences and expectations [14].

In this report, we investigated the relative importance of 16 well-recognized factors in OHCA at the time point of ambulance arrival, and before any interventions (intubation, drugs, mechanical chest compressions, etc.) had been given, by using a machine learning approach that implies building models directly from the data. This creates a data driven approach to variable importance, as well as capturing interactions and non-linear associations automatically. An ever-increasing body of evidence suggests that such machine learning models are superior to regression models [15–18]. Therefore, we set out to examine the relative importance of 16 predictors using machine learning.

## Methods

### Data sources

The data are collected from the Swedish Registry of Cardiopulmonary Resuscitation (SRCR).

Stromsoe et al. have provided a detailed description of SRCR and a validation of the reported data [19]. In short, reporting is comprised of two steps; initial reporting by the Emergency Medical Services (EMS) crews, and follow-up, which is mostly performed by a healthcare provider in the hospital. Online data entry by the EMS has been utilized since late 2007. All EMS units in Sweden report to the registry. We therefore included all 45,067 cases of OHCA recorded during 2008 to 2016, where CPR had been attempted. Information regarding 30-day survival was retrieved from the Cause of Death Registry. Data linkage is virtually complete due to unique personal identification numbers, which are assigned to all Swedes from birth or immigration. We took a closer look into the five strongest and the five weakest factors in predicting survival. Time to CPR and bystander CPR were discussed together and regarded as “early CPR”.

### Predictors of 30-day survival

To employ a data-driven approach, we included all 16 variables available in the Swedish Registry of Cardiopulmonary Resuscitation (SRCR). Some of these variables have previously been reported to be of importance for the chance to survive a cardiac arrest [20]. All variables that are collected in the registry were included. Initial rhythm was categorized as either shockable (ventricular fibrillation [VF] or pulseless ventricular tachycardia [VT]) or non-shockable (pulseless electrical activity (PEA) or asystole). The following time intervals (in minutes) were assessed: time from cardiac arrest (CA) to CPR; time from CA to the 911 call; time from 911 call to Emergency Medical Service (EMS) dispatch; and time from EMS dispatch to EMS arrival at the patients’ side. Place of CA was defined as home, public or non-public places. Non-public places include facilities outside hospital such as nursing homes, dental and primary healthcare facilities, ambulances, but also private work offices. Public places include all other places outside hospital and outside home such as train stations, shopping centers etc. Patients were further divided into those who had OHCA at home or not at home. Whether the CA was witnessed or not, whether CPR was attempted before EMS arrival, whether patients were defibrillated before EMS arrival, and whether patients were defibrillated by the EMS personnel were also dichotomous variables. Defibrillation before ambulance arrival was carried out using AEDs which are widely available in Sweden. Hence, defibrillation before ambulance arrival is in our study synonymous with the use of AEDs. Finally, the calendar year was a categorical variable, ranging from 2008 to 2016. Cause of CA had nine categories: heart disease, drug overdose, accident, pulmonary disease, suffocation, suicide, drowning, or other as reported by the EMS personnel. Thus, the etiology of cardiac arrest was categorized according to the initial assessment of the EMS personnel. Patients were divided into two groups i.e. cardiac etiology or no cardiac etiology.

### Statistical analyses

We used random forest, a machine learning algorithm that has become a standard tool in medical research, to examine the relative importance of 16 clinical predictors assessed during the pre-hospital phase of OHCA [21]. Binary classification models were developed in order to compute the individual importance of the predictors in the models. Since random forest is highly resistant to overfitting, we used 3000 trees for each model, although we observed no material improvement in accuracy beyond 2800 trees. Missing data was imputed using the MICE (Multiple Imputation by Chained Equations) algorithm. We did not impute the following variables: defibrillation (yes/no), time to defibrillation, defibrillation before EMS arrival since these variables refer to a subset of patients, i.e. those who were found in ventricular fibrillation.

We have validated our findings using gradient boosting, and there were no material differences compared with random forest. We performed 10-fold cross validation and obtained an accuracy of 82.1% of the final model.

To elucidate the associations between key predictors (initial rhythm, cause of CA, age, time to CPR, time to EMS arrival) and 30-days survival we used partial dependence plots (PDP). These plots are low-dimensional graphical renderings of the prediction function, making it possible to visualize the association between the predictors and survival, while accounting for all the predictors in the model. PDP is an effective means of explaining the output from machine learning models [22].

### Variable importance

A detailed discussion on variable importance is provided by van der Laan [23]. Briefly, variable importance is a measure of how important each predictor is. The most commonly used importance measure is the permutation accuracy importance. This measure is obtained by randomly permuting each predictor, which disrupts the association between the predictor and the response variable. The new predictions are made with the permuted variable and the remaining non-permuted variables; if the permuted variable was important, the prediction accuracy decreases. Hence, variable importance is measured as the difference in prediction accuracy before and after permuting the variable. These machine learning methods elaborate prediction models and, in passing, automatically quantify the relative importance of all variables included in the model, thus eliminating bias. We used the permutation accuracy importance developed by Strobl et al., which better handles differences in the scales of the predictors (e.g. continuous vs factor variables) as well as correlations between them [24, 25]. The unit for importance is arbitrary. It was normalized to 100 in order to facilitate interpretation. It is derived by quantifying the improvement or loss in predictive accuracy by means of permutation. Data were analyzed using R (R Foundation for Statistical Computing, v 3.6.1).

## Results

### Relative importance of all predictors

The mean age was 68.3 years and women constituted 33.1% of the cohort. A shockable initial rhythm was seen in 21.7% of patients and 5.2% of all patients were defibrillated before EMS arrival. Sixty-five percent of the cases were witnessed. CPR before arrival of EMS was attempted in over half of the total study population, with the vast majority being performed by laymen. The majority of OHCAs occurred at home and two thirds had a cardiac etiology. The median times from collapse to CPR and defibrillation were 3.0 and 15.0 min, respectively. In total, 34 % were defibrillated during the pre-hospital course. Detailed characteristics of the study population and delay times are seen in Table 1.

**Table 1:** Baseline characteristics of 45,067 cases of OHCA

	Overall (***n*** = 45,067)
**Age – n (%)**	
Mean (SD)	68.3 (17.6)
Missing	1631 (3.6%)
**Sex – n (%)**	
Men	30,146 (66.9%)
Women	14,915 (33.1%)
Missing	6 (0.0%)
**Initial rhythm – n (%)**	
Asystole	23,984 (53.2%)
PEA	6844 (15.2%)
VF/VT	9800 (21.7%)
Missing	4439 (9.8%)
**Defibrillated before ambulance arrival – n (%)**	
Defibrillated before EMS arrival	2338 (5.2%)
Not defibrillated before EMS arrival	37,784 (83.8%)
Missing	4945 (11.0%)
**Witnessed arrest – n (%)**	
Non-Witnessed	14,828 (32.9%)
Witnessed	29,428 (65.3%)
Missing	811 (1.8%)
**Bystander-CPR – n (%)**	
CPR by laymen	21,162 (47.0%)
CPR by professional	3402 (7.5%)
No bystander-CPR	16,594 (36.8%)
Missing	3909 (8.7%)
**Place of arrest – n (%)**	
Home	30,970 (68.7%)
Other place	5977 (13.3%)
Public place	8081 (17.9%)
Missing	39 (0.1%)
**Cause of arrest – n (%)**	
Heart disease	28,501 (63.2%)
Overdose	1293 (2.9%)
Accident	1168 (2.6%)
Pulmonary disease	2319 (5.1%)
Suffocation	1143 (2.5%)
Suicide	983 (2.2%)
Drowning	515 (1.1%)
Other	9145 (20.3%)
**Time to 911 call (min)**	
Median (IQR)	2.0 (1.0, 6.0)
Missing	17,941 (39.8%)
**Time to CPR (min)**	
Median (IQR)	3.0 (0.0, 10.0)
Missing	8149 (18.1%)
**Time to defibrillation (min)**	
Median (IQR)	15.0 (9.0, 24.0)
Missing	31,402 (69.7%)
**Time to EMS dispatch**	
Median (IQR)	1.0 (0.0, 2.0)
Missing	4263 (9.5%)
**Time to EMS arrival**	
Median (IQR)	10.0 (6.0, 15.0)
Missing	6399 (14.2%)
**Defibrillation – n (%)**	
Defibrillated	15,390 (34.1%)
Not defibrillated	27,802 (61.7%)
Missing	1875 (4.2%)

*IQR* terquartile range

**Fig. 1:**
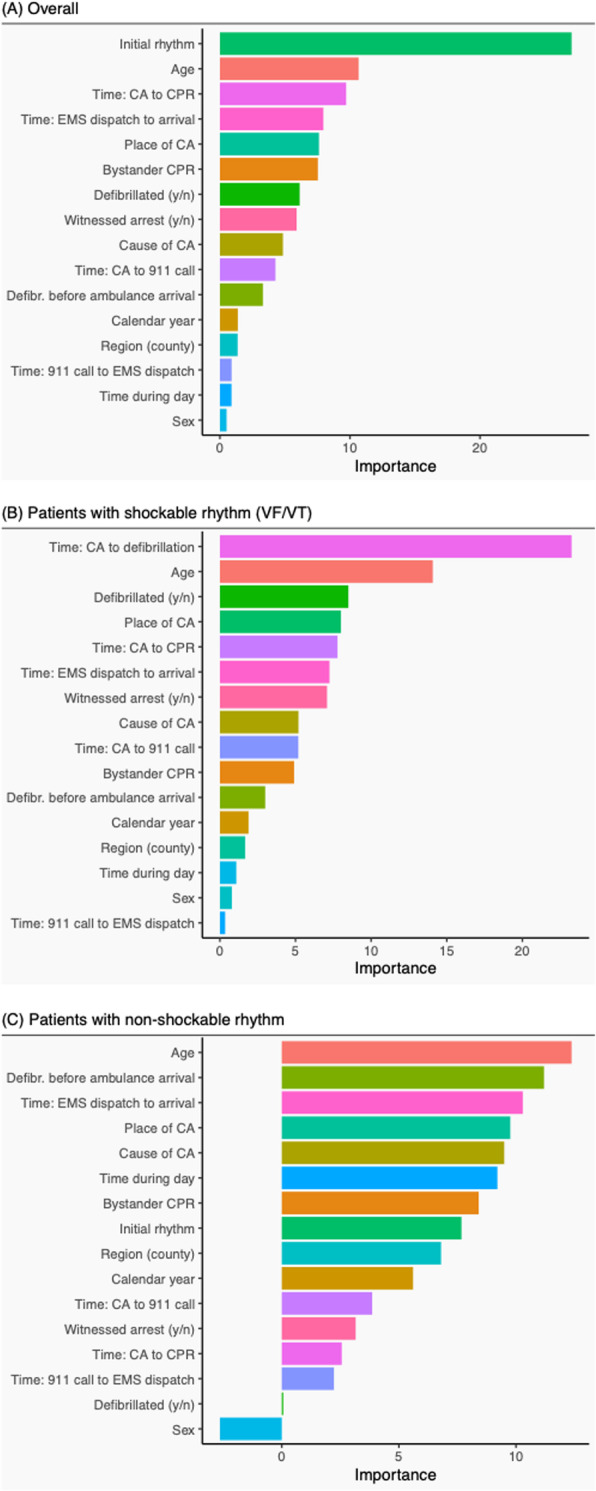
The relative importance of various factors in predicting survival, before any treatment has been given

The relative importance of all predictors is presented in Fig. 1a to c. Overall, the top five factors to predict survival in order of importance were: initial rhythm, age, early CPR (time to CPR and CPR before arrival of EMS), time from EMS dispatch until EMS arrival (EMS response time), and place of CA. The largest difference in importance was noted between initial rhythm and the remaining predictors. The five factors that were of least importance were: sex, time during the day when OHCA took place, time from emergency call to EMS dispatch (procedure time at the dispatch center), region where and calendar year when OHCA took place (Fig. 1a).

Among patients who were found in a shockable rhythm (Fig. 1b), the five most important predictors were: time to defibrillation, age, defibrillation (yes/no), place of cardiac arrest, and time from CA to CPR. The largest difference in importance was noted between time from CA to defibrillation and age. The five least important predictors were the same as for the overall population.

For patients who were found in a non-shockable rhythm, the most important predictors were age, defibrillation before EMS arrival, time to EMS arrival, place of CA and cause of CA. We did not note any particularly strong predictor; importance declined gradually from the most important to the least important predictor (Fig. 1c).

### Association between key predictors and 30-days survival

**Fig. 2:**
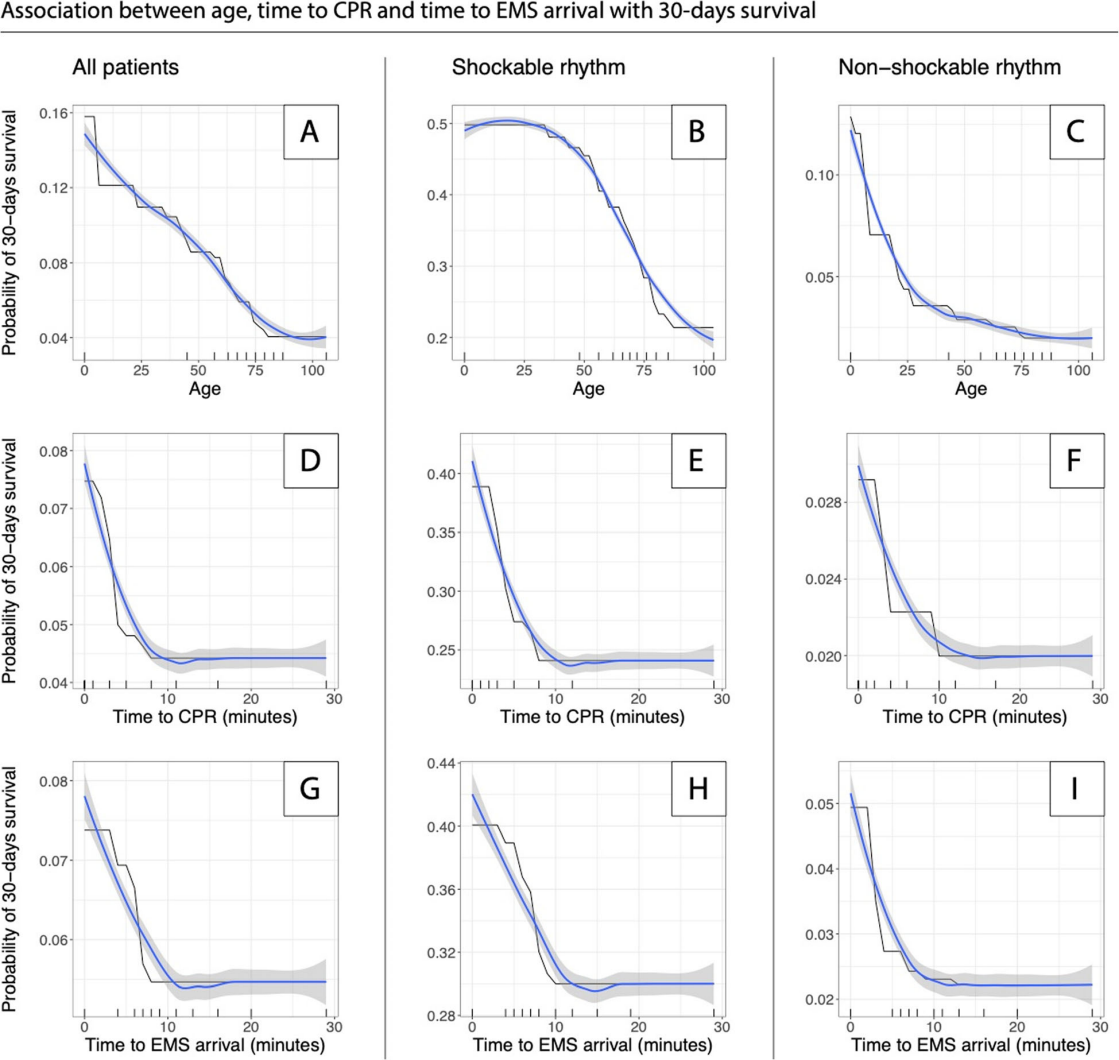
Partial Dependence Plots showing the association between age, time to CPR and time to EMS arrival and 30-days survival. Tick marks on the x-axis represent decile markers

Overall, survival was highest among the youngest patients (Fig. 2a–c). However, the shape of the association curve differed for shockable and non-shockable rhythm, such that survival was relatively stable between 0 and 30 years of age among patients with shockable rhythm, while survival dropped rapidly in the same age range for those with non-shockable rhythm.

The association between time from collapse until start of CPR and 30-days survival was uniform across all three groups (Fig. 2d–f). Overall, survival is reduced by half during the first 10 min from collapse (Fig. 2d). A similar pattern was noted for those who were found in a shockable rhythm (Fig. 2e), and for those who were found in a non-shockable rhythm (Fig. 2f), albeit less pronounced for the latter.

The same pattern was noted for the association between time from EMS dispatch until EMS arrival i.e. EMS response time and 30-days survival.

**Fig. 3:**
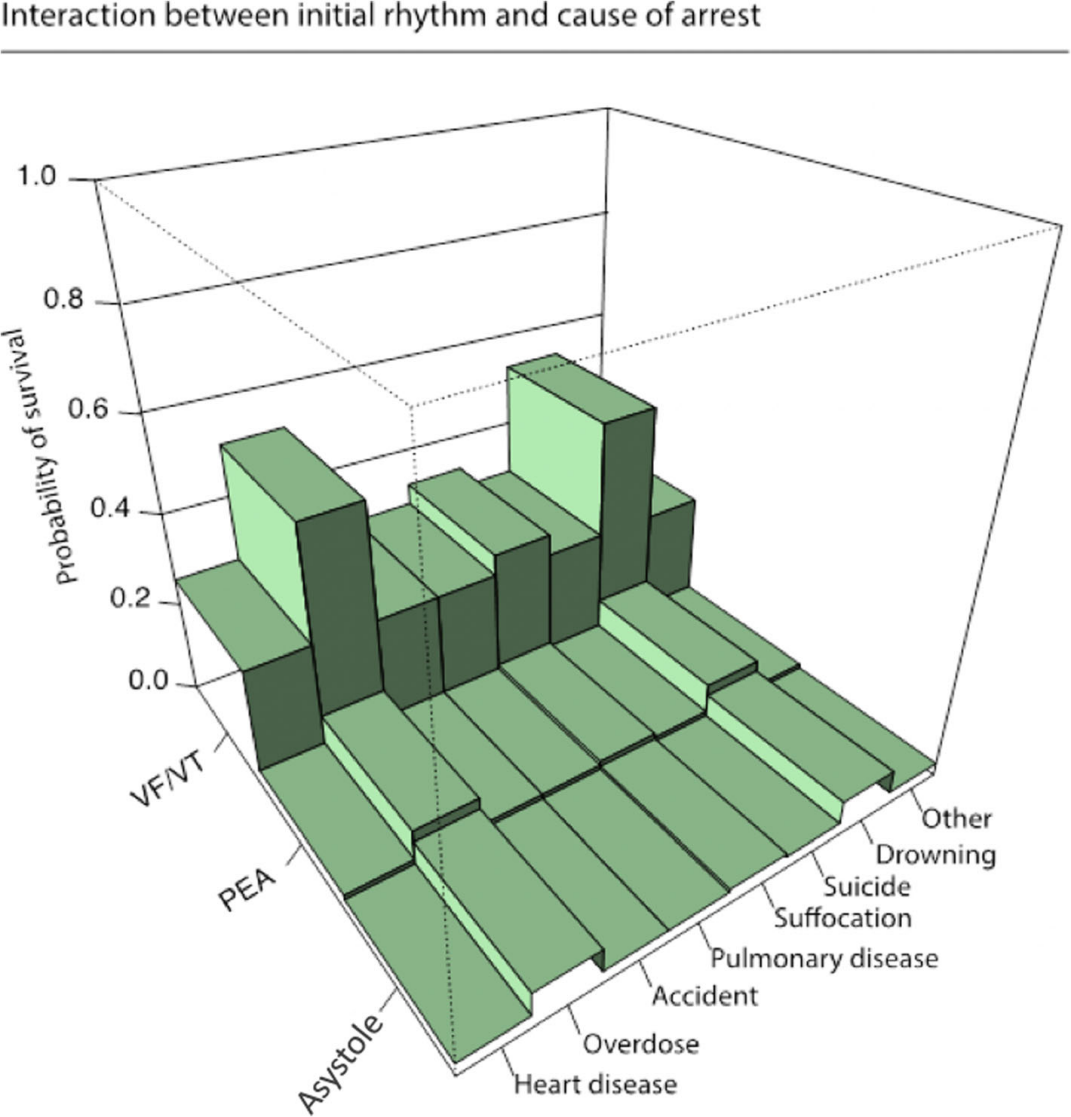
Partial dependence plot showing the interaction between cause of cardiac arrest and initial rhythm

Figure 3 displays the interaction between cause of arrest and initial rhythm and 30-days survival. It is evident that overdose and drowning correlated with better survival across all rhythms, although PEA and asystole consistently confer very low probabilities of survival.

## Discussion

It is important to stress that the primary strength of machine learning is the inherent ability to handle vast amounts of predictors, capture non-linear association, use unstructured/raw data (images, text, video, etc.), and create data driven prediction models without any subject matter knowledge. Machine learning models are currently being deployed in literally every aspect of medical research, with the most promising results obtained using ensemble methods (which was used in this study) and deep learning [15, 26]. Indeed, machine learning will enable clinicians to make decisions and predictions that are superhuman, as evident in recent studies [27, 28]. Resuscitation research stands to benefit from these advances, provided that researchers collect large amounts of useful and multimodal data.

We have examined the relative importance of 16 factors in predicting survival in patients with OHCA, before administration of medications (adrenaline or amiodarone) and interventions (such as mechanical chest compressions or intubation). Using data driven methods, we demonstrate that initial rhythm clearly stands out as the strongest overall predictor of survival. We also note that age was among the top 2 most important predictors in all three analyses, which was evident when viewing the dramatic drop in survival with increasing age (particularly for patients with non-shockable rhythm). We also demonstrate that several predictors which are traditionally considered as important, had little or no importance; these include sex, time when collapse took place, and region. Finally, we demonstrate that delay to CPR and EMS delay times are absolutely crucial, as survival drops in a dramatic fashion during the first 10 min after cardiac arrest.

Some of the overall information listed above is not new and have been reported previously with different statistical methods. Thus, one may say that machine learning confirms previous knowledge about factors of importance for survival after OHCA [2–12, 15]. However, what is new in this article is that we report on the relative importance of different factors for the chance of survival after OHCA, using the least biased method available, to present a hierarchy of importance.

Thus, a major message is that the initial type of arrhythmia is by far the most important factor for the chance of survival after OHCA. The observation that the use of AED is not among the most important factors for survival may be explained by its tight correlation with the initial arrhythmia.

The second most important factor is the patients’ age and the third most important factor is time from collapse until the start of CPR.

One important finding was that despite that an increasing proportion of victims receive CPR before EMS arrival, EMS response time is still among the most important factors for the chance of survival, being more important than whether the collapse was witnessed or not, as well as the assumed etiology behind the cardiac arrest.

Another interesting finding was that among patients who were found in a non-shockable rhythm, age was more important than any other factor for the chance of survival. Furthermore, among patients who were found in a non-shockable rhythm, there was a decrease in survival with increasing age observed over the whole spectrum of ages, even among patients aged less than 18 years. Finally, our data suggest that the relative importance of the type of initial arrhythmia does not seem to be the same across the different etiologies and may thus be more marked when OHCA is caused by drowning and drug overdose. However, these findings need to be confirmed in future studies.

After accounting for the other predictors, sex was not an important predictor of survival in OHCA. Some studies of the importance of gender on outcomes in OHCA have shown that men are more likely to be found in a shockable rhythm, and that men are more likely to survive to 1 month [29, 30]. In contrast, a number of studies have shown that female sex is an independent predictor of an increased chance of survival after OHCA [3, 8, 13, 31]. It is worth mentioning that some of these studies were performed at a time when the survival rate was much lower than today [31].

Thus, due to conflicting results in the previous literature, our data may add important information that the patients’ sex does not seem to be an important factor for the chance of survival after OHCA when other factors are simultaneously considered [32].

Another weak predictor was the time from call to the dispatch center until EMS was dispatched. The FINNRESUSCI Prehospital Study Group have previously shown that a shorter dispatch time may favorably affect survival [33]. Our results might suggest that since OHCA recognition rates are relatively high amongst dispatchers in Scandinavia [33–35], then delay times to EMS dispatch are short, and therefore do not influence survival significantly. Indeed, these delay times are relatively short when related to EMS response times. Also, the effect of delay from emergency call to EMS dispatch may already be mediated by the other delay variables (time from CA to CPR and time from EMS dispatch to EMS arrival). Indeed, this study demonstrated that time to EMS arrival is crucial, as survival drops by 50% during the first 10 min.

### Limitations

Although machine learning algorithms are capable of handling vast number of predictors, the 16 predictors used here are widely regarded as the most relevant predictors (and this was indicated by the accuracy achieved by our model). However, having access to more variables would presumably elucidate additional interesting findings.

Although we used models specifically developed to minimize issues with collinearity, differences in scales, etc., we cannot rule out that variable importance was affected by such factors. Although we limited our analysis to random forest, we did fit gradient boosted trees but noted no material difference in model accuracy.

This is, to our knowledge, one of the first studies of its kind to utilize machine learning to examine the relative importance of various factors in predicting survival after OHCA. Recently, machine learning has been proposed as an appropriate technique to predict outcome after OHCA [36]. More studies, including more variables, are warranted to further delineate the importance of various factors in OHCA.

### Clinical implications

Despite advances in the treatment of OHCA during the last decades, still only a minority will survive the event. Therefore, it is important to develop decision support tools for the EMS staff which can be helpful already before arrival in hospital to make decisions on whether it is meaningful to continue resuscitation or not. The type of data that we report in this study may form the basis for such a tool. Furthermore, our results give indications on which of the links in the chain of survival are particularly important to strengthen even further.

## Conclusions

Using machine learning, we have confirmed previous knowledge about which factors are important for 30-day survival after out of hospital cardiac arrest, and ranked them in order of importance. Initial rhythm is by far the most important predictor of survival, followed by age, time to start of CPR, EMS response time and place of OHCA. In contrast to many previous studies, sex did not appear as an important predictor for outcome. With this technique, we can hopefully create prediction models for outcome in the future, which may support clinicians to adopt an appropriate level of care in relation to the chance of survival.

## Data Availability

The datasets generated during and/or analyzed during the current study are available from the corresponding author on reasonable request.
